# Association Between Admission Pulse Pressure and Long-Term Mortality in Elderly Patients With Type 2 Diabetes Mellitus Admitted for Acute Coronary Syndrome: An Observational Cohort Study

**DOI:** 10.3389/fcvm.2022.855602

**Published:** 2022-05-11

**Authors:** Zijian Wang, Xiaoran Li, Yichun Wang, Boyi Bao, Xiaosong Ding, Hongwei Li, Weiping Li

**Affiliations:** ^1^Department of Cardiology, Cardiovascular Center, Beijing Friendship Hospital, Capital Medical University, Beijing, China; ^2^Department of Cardiology, Beijing Aerospace General Hospital, Beijing, China; ^3^Beijing Key Laboratory of Metabolic Disorder Related Cardiovascular Disease, Beijing, China; ^4^Department of Internal Medicine, Medical Health Center, Beijing Friendship Hospital, Capital Medical University, Beijing, China

**Keywords:** pulse pressure, mortality, elderly, type 2 diabetes mellitus, acute coronary syndrome

## Abstract

**Objective:**

The aim of this study was to assess the effect of pulse pressure (PP) at admission on long-term cardiac and all-cause mortality among elderly patients with type 2 diabetes mellitus (T2DM) admitted for acute coronary syndrome (ACS).

**Methods:**

This is a retrospective observational study. The patients aged at least 65 years with T2DM and ACS from January 2013 to April 2018 were enrolled and divided into 4 groups according to admission PP: <50 mmHg; 50–59 mmHg; 60–69 mmHg, and ≥70 mmHg. Multivariate Cox proportional hazard regression analyses and restricted cubic spline were performed to determine the association between PP and outcomes (cardiac and all-cause death).

**Results:**

A total of 2,587 consecutive patients were included in this cohort study. The mean follow-up time was 39.2 months. The incidences of cardiac death and all-cause death were 6.8% (*n* = 176) and 10.8% (*n* = 280), respectively. After multivariate adjustment in the whole cohort, cardiac and all-cause mortality were significantly higher in PP <50 mmHg group and PP ≥70 mmHg group, compared with PP 50–59 mmHg group. Further analysis in acute myocardial infarction (AMI) subgroup confirmed that PP <50 mmHg was associated with cardiac death [hazard ratios (HR) 2.92, 95% confidence interval (CI) 1.45–5.76, *P* = 0.002] and all-cause death (HR 2.08, 95% CI 1.20–3.58, *P* = 0.009). Meanwhile, PP ≥70 mmHg was associated with all-cause death (HR 1.78, 95% CI 1.05–3.00, *P* = 0.031). However, admission PP did not appear to be a significant independent predictor in unstable angina pectoris (UAP) subgroup. There is a U-shaped correlation between PP and cardiac and all-cause mortality in the whole cohort and UAP subgroup and a J-shaped correlation in the AMI subgroup, both with a nadir at 50–59 mmHg.

**Conclusion:**

In elderly patients with T2DM admitted for ACS, admission PP is an independent and strong predictor for long-term cardiac and all-cause mortality, especially in patients with AMI.

## Introduction

Blood pressure is a powerful, independent predictor of cardiovascular outcome. Currently, many guidelines have recommended the optimal levels of systolic blood pressure (SBP) and diastolic blood pressure (DBP) in various populations. However, the target pulse pressure (PP) has been lacking and controversial. PP, as the difference between SBP and DBP and the pulsatile component of hemodynamics, is considered an indicator of aortic stiffness, i.e., the higher the PP, the stiffer the aorta. In contrast, PP also reflects left ventricular performance and ventricular stroke volume. High PP may play a role in triggering plaque complications at the coronary and cerebral levels (1), and a low PP may help identify patients at risk due to severe left ventricular dysfunction.

Over the past decades, life expectancy, as well as the incidence of type 2 diabetes mellitus (T2DM), has dramatically increased worldwide. It is projected that the proportion of people aged at least 65 years will increase from 12.4% to 19.6% in the United States (2) from the year 2000 to 2030 and 8.2% to 26.9% in China from the year 2010 to 2050 (3, 4). The American Diabetes Association (ADA) recently reported that ~422 million adults were living with diabetes (>90% are T2DM) (5). Besides, as an acute manifestation of ischemic heart disease, acute coronary syndrome (ACS) leads to substantial morbidity and mortality, especially in the geriatric population as well as diabetics.

Although several studies concluded that initial hospital PP was associated with high mortality in overall ACS or patients with post-myocardial infarction (6, 7), the data on the predictive potential of admission PP on long-term outcomes in elderly patients with T2DM admitted for ACS are very limited. Therefore, to address the question of a target for PP in this population, we evaluated the relationships among PP measured at admission and the subsequent cardiac and all-cause mortality over a long-term follow-up period in an observational cohort.

## Methods

### Study Design and Population

**Figure 1:**
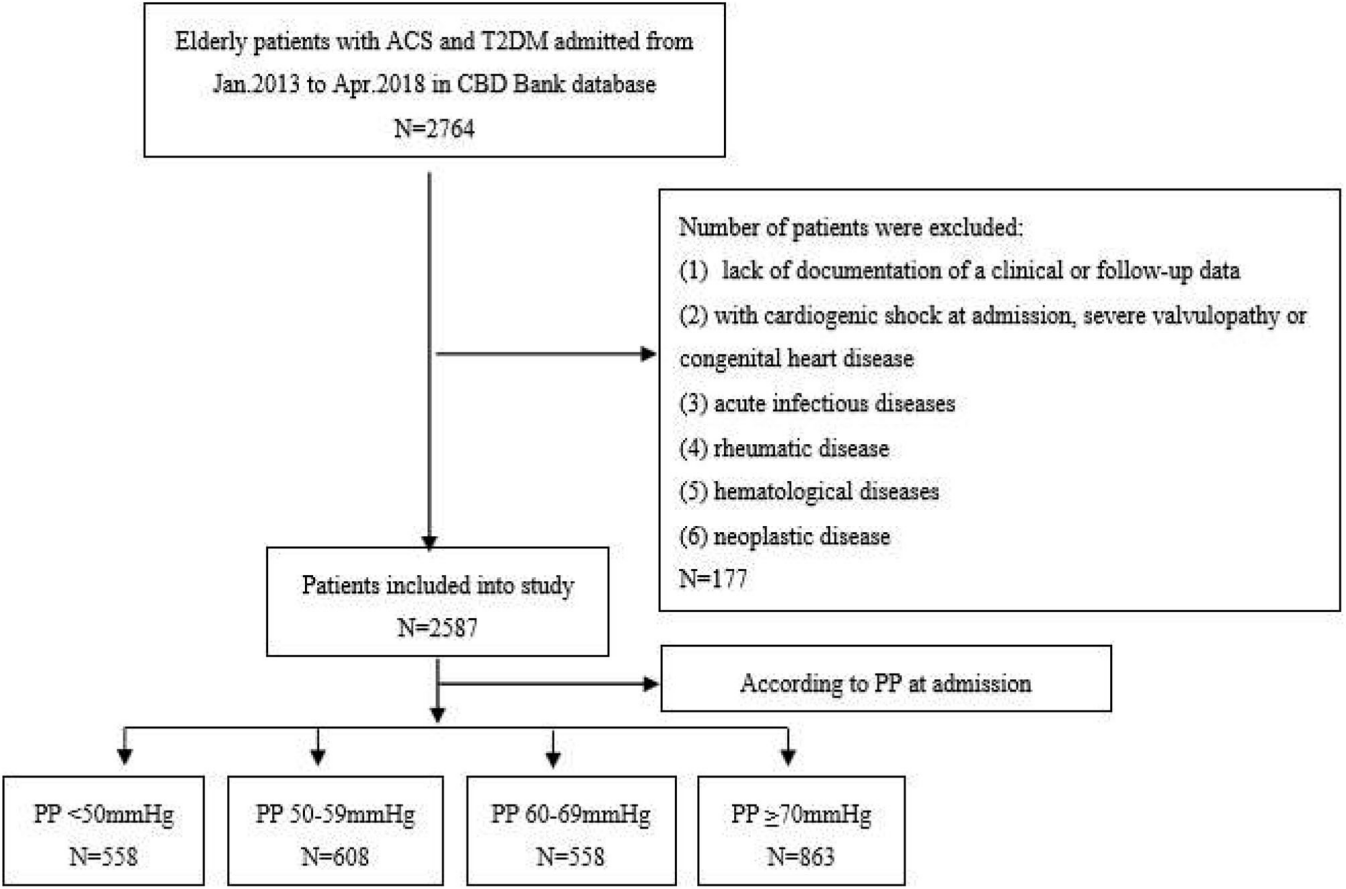
The flowchart of study subject enrollment. CBD, cardiovascular Center Beijing Friendship Hospital Database; ACS, acute coronary syndrome; T2DM, type 2 diabetes mellitus; BP, blood pressure; PP, pulse pressure.

This observational study was based on the Cardiovascular Center Beijing Friendship Hospital Database Bank (CBD Bank). From January 2013 to April 2018, consecutive elderly patients (age ≥ 65 years) diagnosed with T2DM and ACS were included in this study. As shown in Figure 1, among a total of 2,764 patients, 177 were excluded according to the exclusion criteria, which were (1) lack of documentation of a clinical or follow-up data; (2) with cardiogenic shock at admission, severe valvulopathy, or congenital heart disease; (3) with acute infectious diseases, rheumatic disease, hematological diseases (leukemia, lymphoma, and disseminated intravascular coagulation), and neoplastic disease. Finally, 2,587 patients were included in this study. The patients were divided into 4 groups according to the admission PP level: PP <50 mmHg group (*n* = 558), PP 50–59 mmHg (*n* = 608), PP 60–69 mmHg (*n* = 558), or PP ≥ 70 mmHg (*n* = 863). This study was performed in accordance with the principles of the Declaration of Helsinki and approved by the Ethics Committee of Beijing Friendship Hospital affiliated with Capital Medical University. The written informed consent was obtained from all patients.

### Data Collections and Definitions

Patients' demographics, medical history and therapy, laboratory data, and echocardiographic results were collected and verified using an electronic medical recording system. Medical therapy included antiplatelet agents, angiotensin-converting enzyme inhibitor (ACEI)/angiotensin receptor blocker (ARB), beta-blockers, statin, acarbose, metformin, sulfonylurea, insulin, thiazolidinediones, and sodium-dependent glucose transporter 2 (SGLT2) inhibitors.

Admission BP was defined as the measurement immediately obtained at admission to the cardiology department. SBP and DBP were recorded with the patient resting in the supine position for at least 10 min, using the standard mercury sphygmomanometer. Measurements were made twice at 15-min intervals, and the average value was recorded as the final reading. PP was defined as SBP minus DBP. Blood samples were collected at admission and after 12 h of overnight fasting, and laboratory measurements were made at the Central Laboratory of Beijing Friendship Hospital. The estimated glomerular filtration rate (eGFR) was calculated based on the Modification of Diet in Renal Disease formula (8). Echocardiography was performed within 48 h of hospitalization using a VIVID 7 (General Electric Medical Systems, Horten, Norway).

The criteria for T2DM include: (1) previously diagnosed T2DM under treatment of antidiabetic medication; (2) the typical symptoms of DM with a fasting plasma glucose (FPG) ≥7.0 mmol/L, and/or random blood glucose (RBG) ≥11.1 mmol/L, and/or 2-h plasma glucose level after oral glucose tolerance test (OGTT) ≥11.1 mmol/L (9). ACS was composed of ST-segment elevation myocardial infarction (STMI), non-ST-segment elevation myocardial infarction (NSTEMI), and unstable angina pectoris (UAP), definitions of which were determined by appropriate guidelines (10, 11). Myocardial infarction was defined as having symptoms of ischemia with new ST-segment changes and an increase of cardiac troponin I (cTnI) or troponin T (cTnT) values above the 99th percentile upper reference limit (URL). UAP was diagnosed as ischemic symptoms at rest, or exacerbated or new-onset symptoms with transient ischemic ST-segment shifts, and without the release of myocardial enzymes related to myocardial necrosis. Cardiogenic shock was defined as hypotension (an SBP of <90 mmHg for at least 30 min or the need for supportive measures to maintain an SBP of at least 90 mmHg) and evidence of end-organ hypoperfusion (a heart rate of ≥60 beats/min and cool extremities or a urine output of <30 ml/h), or Killip class IV (12).

### Study Outcomes and Follow-Up

All patients were followed up for at least 1-year or until death with a mean (SD) of 39.2 (18.6) months. The primary outcome was cardiac death during hospitalization and over the follow-up period. All-cause death was a secondary outcome. Cardiac death was defined as any death with a demonstrable cardiac cause or any death that is not clearly attributable to a noncardiac cause. The clinical outcomes were collected and recorded during hospitalization and follow-up visits for each participant at 1, 3, and 6 months and every year after discharge until death, by contacting the patient or his/her family and/or reviewing hospital records.

### Statistical Analysis

Data are expressed as mean ± standard deviation (SD) or median (interquartile ranges) for continuous variables and as numbers (percentages) for categorical variables. Intergroup comparison of continuous variables between PP groups with PP 50–59 mmHg group was performed using the Bonferroni or Kruskal–Wallis H-test for continuous variables and Pearson chi-square test or Fisher's exact test for categorical variables, where appropriate. We used restricted cubic splines with three knots at the 10, 50, and 90th centiles to flexibly model nonlinear associations between admission PP and cardiac or all-cause death (13, 14). Cox proportional hazard regression model with PP 55–59 mmHg as reference was used, and baseline variables that were significantly different among 4 PP groups and clinically relevant were entered into the multivariate model. In this model, the adjusted covariates included the diagnosis of acute myocardial infarction (AMI), age, male sex, previous MI, chronic heart failure, hypertension, duration of diabetes (<5 years, 5–9 years, 10–14 years, 15–19 years, and ≥20 years), current smoking, heart rate, medical therapies before admission including antiplatelet agents, ACEI/ARB, metformin and insulin, fasting glucose, HbA1c, low-density lipoprotein cholesterol (LDL-C), eGFR, cTnI peak, left ventricular eject fraction (LVEF), in-hospital treatments of antiplatelet agents, ACEI/ARB, statin, and metformin. The cumulative incidence of mortality was estimated using the adjusted multivariate Cox proportional hazard regression curve. Statistical analyses were performed using the Statistical Package for Social Sciences, version 23 (IBM Inc., Armonk, NY, USA) software and STATA statistical software (Version 14, STATA Corp, College Station, TX, USA). Figures were generated using GraphPad Prism version 6.0d (GraphPad Software). A two-tailed *P*-value < 0.05 was considered statistically significant.

## Results

### Baseline Characteristics of Patients

**Figure 2:**
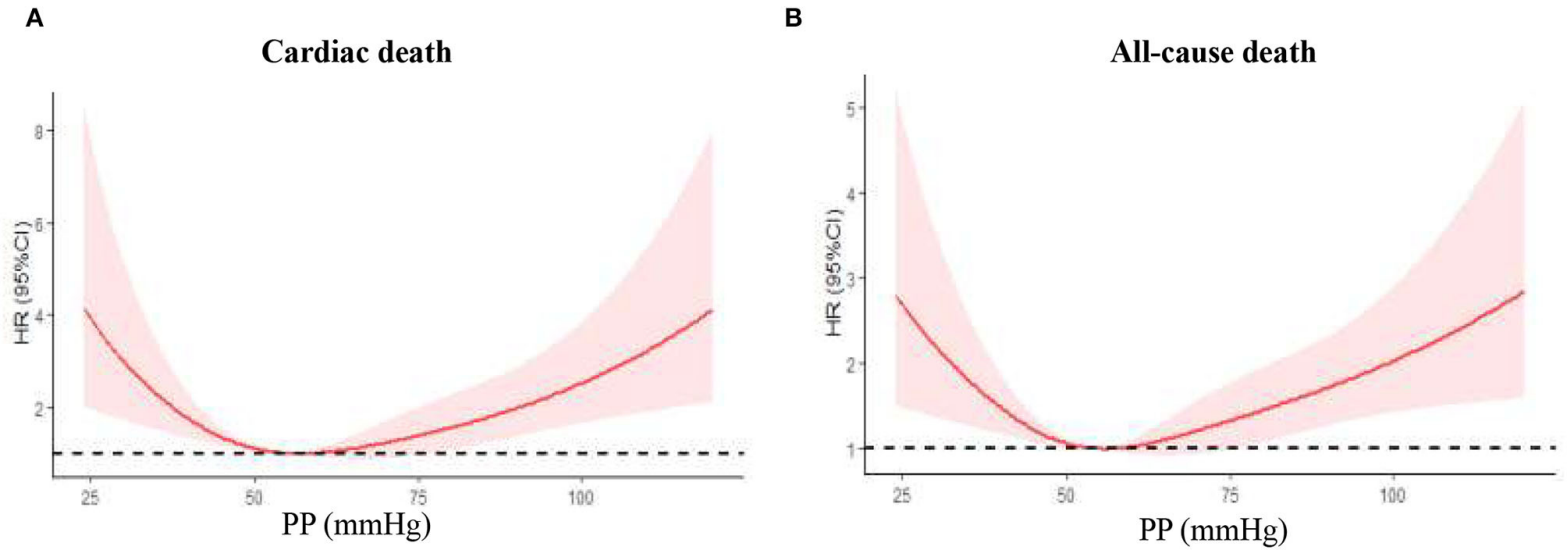
Nonlinear relation between continuous admission PP and cardiac death or all-cause death was assessed using restricted cubic splines. PP, pulse pressure. **(A)** Cardiac death in the whole cohort; **(B)** all-cause death in the whole cohort.

The study cohort constituted 2,587 patients aged at least 65 years, of whom 715 (27.7%) with AMI and 1,872 (72.3%) with UAP. The mean (SD) age of the cohort was 74.2 (6.2) years, and 48% were men. During the mean 39.2-month follow-up, 176 (6.8%) patients had cardiac death, and 280 (10.8%) patients had all-cause death. As shown in Figure 2, there was a U-curve relationship between admission PP and cardiac and all-cause mortality, with a nadir at 50–59 mmHg. Baseline characteristics of the patients in the whole cohort and in groups stratified by the categories of PP were presented in Table 1. Compared with patients in PP 50–59 mmHg group, patients in the lowest PP group (<50 mmHg) had higher percentages of AMI at admission and previous chronic heart failure, lower hypertension history, prior treatment of ACEI/ARB and LVEF, and higher levels of HbA1c and cTnI peak; whereas patients in the highest PP group (≥70 mmHg) had older age, higher rate of AMI, female gender, previous coronary artery disease (CAD) and hypertension history, longer duration of diabetes, higher prior treatment of ACEI/ARB and insulin, lower prior treatment of metformin, higher fasting glucose and LDL-C values, and lower eGFR. As for in-hospital treatment, patients with the lowest PP (<50 mmHg) showed lower ratios of antiplatelet agents, ACEI/ARB, and statin; whereas patients with the highest PP (≥70 mmHg) had higher ratios of ACEI/ARB and metformin. Meanwhile, the proportions of PCI/CABG and other medical therapies among all groups were similar.

**Table 1:** Baseline clinical characteristics of patients stratified by admission PP categories.

	**Total (*n* = 2,587)**	**PP <50 mmHg (*n =* 558)**	***P*^a^ value**	**PP 50-59 mmHg (*n =* 608)**	**PP 60-69 mmHg (*n =* 558)**	***P*^a^ value**	**PP ≥70 mmHg (*n =* 863)**	***P*^a^ value**
Diagnosis of AMI, n(%)	715 (27.7)	163 (29.3)	0.043	146 (24.1)	137 (24.6)	0.825	269 (31.2)	0.002
Male gender, n(%)	1241 (48.0)	307 (55.1)	0.150	308 (50.7)	269 (48.3)	0.385	357 (41.4)	0.002
Age, year	74.2 ± 6.2	73.3 ± 6.1	0.883	73.2 ± 6.1	73.4 ± 5.8	0.109	75.4 ± 6.2	<0.001
BMI, Kg/m^2^	25.6 ± 3.5	25.3 ± 3.5	0.075	25.8 ± 3.5	25.6 ± 3.4	0.358	25.7 ± 3.5	0.320
Medical history								
CAD, n(%)	1882 (72.7)	402 (72.1)	0.097	438 (72.0)	395 (70.8)	0.024	647 (75.0)	0.016
Previous MI, n(%)	364 (14.1)	90 (16.2)	0.916	95 (15.7)	64 (11.5)	0.030	115 (13.4)	0.185
PCI, n(%)	754 (29.1)	169 (30.3)	0.451	197 (32.3)	155 (27.8)	0.346	233 (27.0)	0.176
CABG, n(%)	146 (5.6)	37 (6.6)	0.251	31 (5.1)	22 (3.9)	0.396	56 (6.5)	0.252
CHF, n(%)	53 (2.1)	20 (3.6)	0.032	11 (1.9)	9 (1.7)	0.816	13 (1.6)	0.689
Hypertension, n(%)	2167 (83.8)	400 (71.7)	<0.001	498 (82.1)	482 (86.4)	0.042	787 (91.2)	<0.001
Dyslipidemia, n(%)	1220 (47.2)	282 (50.6)	0.363	290 (47.7)	256 (45.9)	0.515	392 (45.5)	0.372
Duration of diabetes, years	12.6 ± 8.3	11.6 ± 7.9	0.685	11.8 ± 8.0	12.5 ± 8.5	0.142	13.8 ± 8.5	<0.001
Current smoker, n(%)	961 (37.1)	158 (41.4)	0.971	224 (39.4)	227 (38.6)	0.712	352 (33.6)	0.009
SBP, mmHg	134 ± 20	112 ± 11	<0.001	122 ± 11	131 ± 11	<0.001	150 ± 17	<0.001
DBP, mmHg	73 ± 11	74 ± 10	0.781	73 ± 10	72 ± 11	0.056	72 ± 11	0.030
HR, bpm	72 ± 13	75 ± 15	0.246	72 ± 13	72 ± 14	0.406	71 ± 12	0.007
Medical therapies before admission								
Antiplatelet agents, n(%)	1,335 (51.7)	286 (51.3)	0.231	345 (56.8)	289 (51.8)	0.931	415 (48.1)	0.179
ACEI/ARB, n(%)	1,004 (38.9)	171 (30.7)	0.021	227 (37.4)	227 (40.7)	0.231	379 (44.0)	0.010
Beta-blocker, n(%)	557 (21.6)	118 (21.2)	0.373	140 (23.1)	118 (21.2)	0.405	181 (21.0)	0.317
Statin, n(%)	852 (33.0)	181 (32.5)	0.712	202 (33.3)	200 (35.9)	0.362	269 (31.2)	0.067
Acarbose, n(%)	1,303 (50.4)	286 (51.3)	0.513	301 (49.6)	284 (50.9)	0.616	432 (50.1)	0.811
Metformin, n(%)	730 (28.3)	163 (29.3)	0.673	185 (30.5)	170 (30.5)	0.973	212 (24.6)	0.015
Sulfonylurea, n(%)	595 (23.0)	116 (20.8)	0.499	137 (22.6)	138 (24.8)	0.365	204 (23.7)	0.608
Insulin, n(%)	920 (35.6)	183 (32.8)	0.558	188 (31.0)	197 (35.4)	0.127	352 (40.8)	<0.001
Thiazolidinediones, n(%)	188 (7.3)	41 (7.4)	0.844	43 (7.1)	41 (7.3)	0.851	63 (7.3)	0.973
SGLT2 inhibitors, n(%)	58 (2.2)	13 (2.3)	0.963	14 (2.3)	12 (2.2)	0.864	19 (2.2)	0.901
Laboratoy values								
Fasting glucose, mmol/L	7.38 ± 2.66	7.42 ± 2.66	0.137	7.2 ± 2.37	7.21 ± 2.64	0.902	7.59 ± 2.86	0.006
Admission glucose, mmol/L	9.53 (7.30,12.85)	9.65 (7.37,13.13)	0.243	9.58 (7.35,12.46)	9.51 (7.22,12.62)	0.482	9.50 (7.34,13.05)	0.952
HbA1c, %	7.41 ± 1.33	7.56 ± 1.41	0.002	7.28 ± 1.23	7.34 ± 1.4	0.475	7.43 ± 1.3	0.058
TG, mmol/L	1.60 ± 1.14	1.66 ± 0.99	0.124	1.56 ± 1.02	1.56 ± 1.05	0.955	1.60 ± 1.34	0.452
TC, mmol/L	4.12 ± 1.07	4.12 ± 1.01	0.288	4.05 ± 0.96	4.21 ± 1.08	0.010	4.14 ± 1.16	0.102
HDL-C, mmol/L	1.09 ± 0.28	1.08 ± 0.28	0.847	1.08 ± 0.27	1.10 ± 0.29	0.276	1.11 ± 0.28	0.068
LDL-C, mmol/L	2.27 ± 0.73	2.22 ± 0.71	0.998	2.22 ± 0.69	2.22 ± 0.74	0.936	2.36 ± 0.75	<0.001
eGFR, ml/min/1.73 m^2^	66.5 ± 19.5	68.5 ± 19.6	0.976	68.7 ± 18.1	67.1 ± 18.9	0.188	63.1 ± 20.3	<0.001
cTnI peak, ng/mL	1.700 (0.002,5.000)	1.960 (0.002,5.440)	0.015	1.100 (0.002,4.422)	1.700 (0.002,5.000)	0.188	1.800 (0.016,5.000)	0.287
LVEF, %	62.5 ± 10.3	59.7 ± 11.6	<0.001	63.0 ± 10.1	63.4 ± 9.9	0.529	63.3 ± 9.4	0.604
Multi-vessel disease, n(%)	1,544 (59.7)	485 (58.8)	0.328	211 (61.9)	349 (62.5)	0.843	499 (57.8)	0.196
In-hospital treatment								
PCI/CABG	1,032 (39.9)	213 (38.2)	0.453	246 (40.4)	240 (43.0)	0.362	333 (38.6)	0.486
Antiplatelet agents, n(%)	2,332 (90.1)	480 (86.3)	0.002	557 (91.5)	514 (92.1)	0.708	781 (90.5)	0.541
ACEI/ARB, n(%)	1,524 (58.9)	259 (46.7)	<0.001	354 (58.4)	323 (57.7)	0.932	588 (68.0)	<0.001
Beta-blocker, n(%)	1,767 (68.6)	376 (67.2)	0.150	435 (71.1)	381 (68.2)	0.248	575 (66.6)	0.06
Statin, n(%)	2,206 (85.0)	443 (79.5)	<0.001	542 (89.3)	480 (85.9)	0.150	741 (85.9)	0.094
Acarbose, n(%)	1373 (53.1)	300 (53.9)	0.575	318 (52.2)	285 (51.1)	0.696	470 (54.5)	0.212
Metformin, n(%)	728 (28.1)	142 (25.5)	0.658	162 (26.5)	159 (28.5)	0.918	265 (30.6)	0.004
Sulfonylurea, n(%)	496 (19.2)	91 (16.3)	0.336	113 (18.6)	127 (22.8)	0.068	165 (19.1)	0.786
Insulin, n(%)	726 (28.1)	140 (25.1)	0.578	162 (26.6)	159 (28.5)	0.472	265 (30.8)	0.084
Thiazolidinediones, n(%)	162 (6.3)	34 (6.1)	0.924	38 (6.2)	39 (7.0)	0.598	51 (5.9)	0.797
SGLT2 inhibitors, n(%)	70 (2.7)	16 (2.9)	0.797	16 (2.6)	15 (2.7)	0.949	23 (2.7)	0.965

*Values are presented as mean ± SD, median (IQR), or number (%)*.

*PP, pulse pressure; AMI, acute myocardial infarction; CAD, coronary artery disease; MI, myocardial infarction; CHF, chronic heart failure; SBP, systolic blood pressure; DBP, diastolic blood pressure; HR, heart rate; BMI, body mass index; ACEI, angiotensin-converting enzyme inhibitor; ARB, angiotensin receptor blocker; SGLT2, sodium-dependent glucose transporters 2; HbA1c, glycosylated hemoglobin; TC, total cholesterol; TG, total triglyceride; LDL-C, low-density lipoprotein cholesterol; HDL-C, high-density lipoprotein cholesterol; eGFR, estimated glomerular filtration rate; cTnI, cardiac troponin I; LVEF, left ventricular ejection fraction; PCI, percutaneous coronary intervention; CABG, coronary artery bypass graft*.

*P^a^ values are for comparisons with PP 50–59 mmHg*.

### Cardiac and All-Cause Mortality During Follow-Up

As shown in Table 2, among the whole cohort, patients with lowest PP (<50 mmHg) had the highest cardiac death [9.0% (<50 mmHg) vs. 4.1% (50–59 mmHg), *P* = 0.001] and all-cause death [12.9% (<50 mmHg) vs. 7.6% (50–59 mmHg), *P* = 0.003]. No significant differences in any mortality were found between patients in PP 50–59 mmHg group and those in PP 60–69 mmHg group. In addition, patients with highest PP (≥70 mmHg) had higher cardiac death (1.6% vs. 0.2%, *P* = 0.029; 1.9% vs. 0.3%, *P* = 0.030) and all-cause death (8.1% vs. 4.1%, *P* = 0.003; 12.7% vs. 7.6%, *P* = 0.002) when compared with patients in PP 50–59 mmHg group and higher all-cause death (12.7% vs. 9.3%, *P* = 0.042) when compared with patients in PP 60–69 mmHg group.

**Table 2:** Follow-up mortality according to admission PP categories in whole cohort and subgroups.

**Whole cohort**	**Total (*n =* 2587)**	**PP <50 mmHg (*n =* 558)**	***P*^a^ value**	**PP 50-59 mmHg (*n =* 608)**	**PP 60-69 mmHg (*n =* 558)**	***P*^a^ value**	**PP ≥70mmHg (*n =* 863)**	***P*^a^ value**
Cardiac death, n(%)	176 (6.8)	50 (9.0)	0.001	25 (4.1)	31 (5.6)	0.325	70 (8.1)	0.003
All-cause death, n(%)	280 (10.8)	72 (12.9)	0.003	46 (7.6)	52 (9.3)	0.331	110 (12.7)	0.002
**UAP subgroup**	**Total** **(*****n** **=*** **1872)**	**PP** **<** **50 mmHg** **(*****n** **=*** **394)**	***P*^a^ value**	**PP 50-59 mmHg (*****n** **=*** **463)**	**PP 60-69 mmHg (*****n** **=*** **421)**	***P*^a^ value**	**PP** **≥70 mmHg (*****n** **=*** **594)**	***P*^a^ value**
Cardiac death, n(%)	69 (3.7)	13 (3.3)	0.831	14 (3.0)	14 (3.3)	0.812	28 (4.7)	0.148
All-cause death, n(%)	140 (7.5)	29 (7.4)	0.397	27 (5.8)	32 (7.6)	0.318	52 (8.8)	0.068
**AMI subgroup**	**Total** **(*****n** **=*** **715)**	**PP** **<** **50 mmHg (*****n** **=*** **163)**	***P*^a^ value**	**PP 50-59 mmHg (*****n** **=*** **146)**	**PP 60-69 mmHg (*****n** **=*** **137)**	***P*^a^ value**	**PP** **≥70 mmHg (*****n** **=*** **269)**	***P*^a^ value**
Cardiac death, n(%)	107 (15.0)	37 (22.7)	<0.001	11 (7.5)	17 (12.4)	0.247	42 (15.6)	0.027
All-cause death, n(%)	140 (19.6)	43 (26.4)	0.003	19 (13.0)	20 (14.6)	0.736	58 (21.6)	0.035

*PP, pulse pressure; UAP, unstable angina pectoris; AMI, acute myocardial infarction*.

*P^a^ values are for comparisons with PP 50–59 mmHg*.

In patients with UAP, 140 (7.5%) died during follow-up. No significant differences in mortality were found among all groups, although patients with highest PP (≥70 mmHg) had the highest cardiac death (4.7%) and all-cause death (8.8%). However, the different association of admission PP and mortality in patients with AMI was observed. Patients with lowest PP (<50 mmHg) had the highest cardiac death [22.7% (<50 mmHg) vs. 7.5% (50–59 mmHg), *P* < 0.001; 12.4% (60–69 mmHg), *P* = 0.012; and 15.6% (≥70 mmHg), *P* = 0.044] and all-cause death [26.4% (<50 mmHg) vs. 13.0% (50–59 mmHg), *P* = 0.003; 14.6% (60–69 mmHg), *P* = 0.01], respectively. In addition, patients with highest PP (≥70 mmHg) had higher follow-up cardiac and all-cause death when compared with patients in PP 50–59 mmHg group (15.6% vs. 7.5%, *P* = 0.027; 21.6% vs. 13.0%, *P* = 0.035).

### Admission PP Predicted the Occurrence of Long-Term Cardiac and All-Cause Death in the Whole Cohort

**Figure 3:**
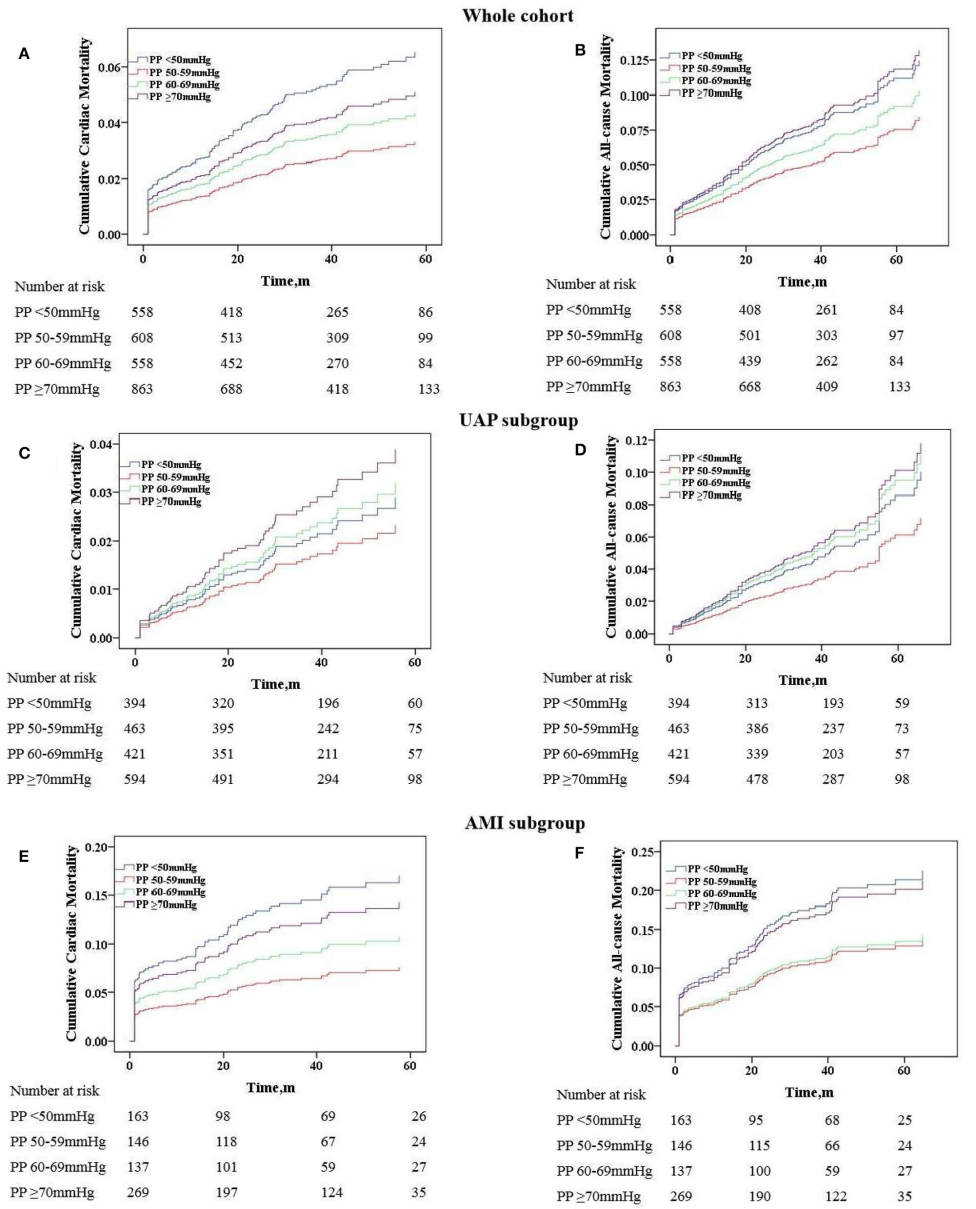
Adjusted cumulative follow-up mortality by COX proportional hazard model. **(A)** Cardiac mortality in the whole cohort; **(B)** all-cause mortality in the whole cohort; **(C)** cardiac mortality in the UAP subgroup; **(D)** all-cause mortality in the UAP subgroup; **(E)** cardiac mortality in the AMI subgroup; **(F)** all-cause mortality in the AMI subgroup. PP, pulse pressure; UAP, unstable angina pectoris; AMI, acute myocardial infarction.

Univariate and multivariate Cox proportional hazard regression analyses in the predictive value of PP for cardiac and all-cause death are presented in Table 3. In this model, the PP 50–59 mmHg group was considered the reference group. Notably, after adjusting for the potential confounding factors, patients with lowest PP (<50 mmHg) had higher risk for follow-up cardiac death (HR 1.89, 95% CI 1.16–3.06, *P* = 0.010) and all-cause death (HR 1.62, 95% CI 1.12–2.35, *P* = 0.011), whereas patients with highest PP (≥70 mmHg) exhibited a similar significantly increased risk for follow-up cardiac death (HR 1.73, 95% CI 1.09–2.76), *P* = 0.020) and all-cause death (HR 1.59, 95% CI 1.12–2.35, *P* = 0.010). Figure 3 shows the continuous relationships between PP and outcomes assessed by adjusted restrict cubic splines. A U-shaped correlation between admission PP and cardiac death or all-cause death during follow-up was observed in the whole cohort and UAP subgroup, with a nadir at 50–59 mmHg, and a J-shaped correlation in the AMI subgroup with the same nadir.

**Table 3:** Univariate and multivariate Cox proportional hazard regression of admission PP for predicting cardiac death and all-cause death in whole cohort and subgroups.

	**Total (*****n** **=*** **2587)**				**UAP (*****n** **=*** **1872)**				**AMI (*****n*** **=** **715)**			
	**Univariate regression**		**Multivariable regression**		**Univariate regression**		**Multivariable regression**		**Univariate regression**		**Multivariable regression**	
	**HR(95%CI)**	***P* value**	**HR(95%CI)**	***P* value**	**HR(95%CI)**	***P* value**	**HR(95%CI)**	***P* value**	**HR(95%CI)**	***P* value**	**HR(95%CI)**	***P* value**
**Cardiac death**									
PP <50 mmHg	2.30 (1.43–3.73)	0.001	1.89 (1.16–3.06)	0.010	1.14 (0.56–2.42)	0.738	1.026 (0.48–2.20)	0.948	3.30 (1.68–6.46)	0.001	2.92 (1.45–5.76)	0.002
PP 50–59 mmHg	1 (reference)	/	1 (reference)	/	1 (reference)	/	1 (reference)	/	1 (reference)	/	1 (reference)	/
PP 60–69 mmHg	1.36 (0.82–2.35)	0.226	1.36 (0.80–2.30)	0.258	1.13 (0.54–2.38)	0.740	1.23 (0.58–2.60)	0.593	1.68 (0.79–3.59)	0.178	1.62 (0.78–3.46)	0.213
PP ≥70 mmHg	2.05 (1.30–3.23)	0.002	1.73 (1.09–2.76)	0.020	1.62 (0.85–3.07)	0.141	1.58 (0.81–3.06)	0.179	2.14 (1.10–4.16)	0.250	1.93 (0.99–3.77)	0.540
**All–cause death**												
PP <50 mmHg	1.83 (1.26–2.65)	0.001	1.62 (1.12–2.35)	0.011	1.34 (7.93–2.26)	0.274	1.675 (0.94–2.98)	0.078	2.23 (1.30–3.83)	0.004	2.08 (1.20–3.58)	0.009
PP 50–59 mmHg	1 (reference)	/	1 (reference)	/	1 (reference)	/	1 (reference)	/	1 (reference)	/	1 (reference)	/
PP 60–69 mmHg	1.27 (0.86–1.90)	0.229	1.21 (0.819–1.81)	0.330	1.38 (0.83–2.31)	0.217	0.99 (0.51–1.90)	0.962	1.15 (0.61–2.15)	0.670	1.12 (0.60–2.01)	0.732
PP ≥70 mmHg	1.76 (1.25–2.49)	0.001	1.59 (1.12–2.25)	0.010	1.57 (0.99–2.50)	0.057	1.45 (0.85–2.48)	0.164	1.72 (1.02–2.88)	0.041	1.78 (1.05–3.00)	0.031

*Multivariate analysis adjusted for AMI, age, male sex, previous MI, CHF, hypertension, duration of diabetes, current smoking, heart rate, medical therapies before admission including antiplatelet agents, ACEI/ARB, metformin and insulin, fasting glucose, HbA1c, LDL-C, eGFR, cTnI peak, LVEF, in-hospital treatments of antiplatelet agents, ACEI/ARB, statin, and metformin*.

*PP, pulse pressure; UAP, unstable angina pectoris; AMI, acute myocardial infarction; MI, myocardial infarction; CHF, chronic heart failure; ACEI, angiotensin-converting enzyme inhibitor; ARB, angiotensin receptor blocker; HbA1c, glycosylated hemoglobin; LDL-C, low-density lipoprotein cholesterol; eGFR, estimated glomerular filtration rate; cTnI, cardiac troponin I; LVEF, left ventricular ejection fraction*.

*P-values are for comparisons with PP 50–59 mmHg*.

### Analyses of Admission PP on Predictive Value for Mortality in UAP and AMI Subgroups

**Figure 4:**
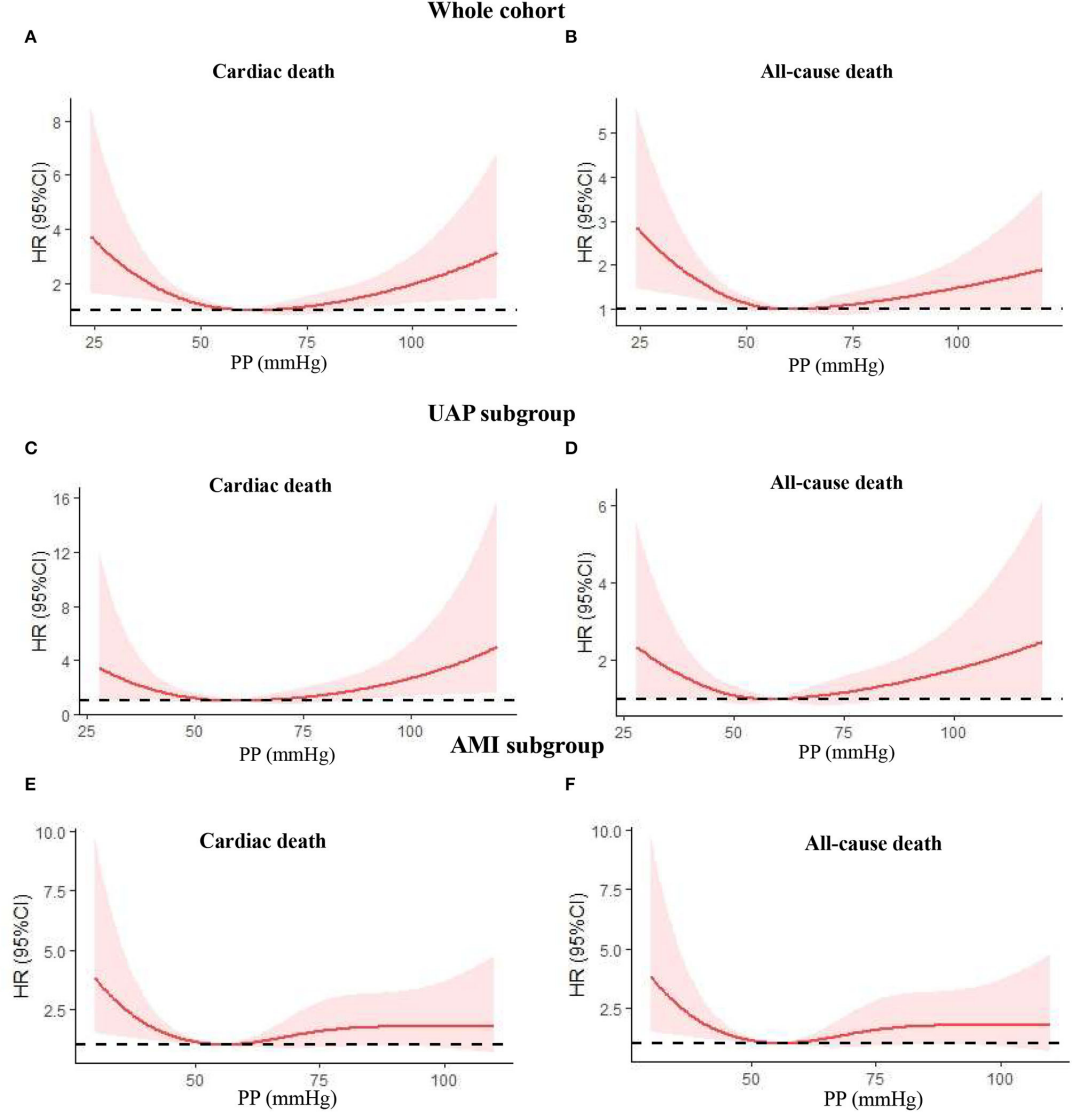
Nonlinear relation between continuous admission PP and cardiac death or all-cause death was assessed using adjusted restricted cubic splines. **(A)** Cardiac death in the whole cohort; **(B)** all-cause death in the whole cohort; **(C)** cardiac death in the UAP subgroup; **(D)** all-cause death in the UAP subgroup; **(E)** cardiac death in the AMI subgroup; **(F)** all-cause death in the AMI subgroup. PP, pulse pressure; UAP, unstable angina pectoris; AMI, acute myocardial infarction; HR, hazard ratio; CI, confidence interval.

As we know, the physiological mechanism and prognosis of UAP and AMI are not completely consistent. Therefore, we further analyzed the predictive value of PP for mortality in these two subgroups. As shown in Table 3, after adjusting for the potential confounding factors in the UAP subgroup, PP no longer independently predicted follow-up cardiac and all-cause death, although patients with the lowest PP (<50 mmHg) showed a trend toward increased risk for follow-up all-cause death (HR 1.675, 95% CI 0.94–2.98, *P* = 0.078). Nevertheless, PP remained to have a predictive value for mortality after adjustment in the AMI subgroup. When compared with patients in the PP 50–59 mmHg group, patients in PP <50 mmHg group still had higher risk for follow-up cardiac death (HR 2.92, 95% CI 1.45–5.76, *P* = 0.002) and all-cause death (HR 2.08, 95% CI 1.20–3.58, *P* = 0.009), respectively. In addition, only patients with highest PP (≥70 mmHg) had higher risk for all-cause death (HR 1.78, 95% CI 1.05–3.00, *P* = 0.031). Figure 4 illustrates the adjusted cumulative follow-up mortality risk by COX proportional hazard model in different PP groups.

## Discussion

In this study, we evaluated the prognostic value of PP at admission for cardiac and all-cause mortality in elderly patients with T2DM admitted for ACS in a real-world setting, when receiving modern guideline-based management. Our main findings include: (1) there is a U-shaped correlation between admission PP and long-term cardiac and all-cause death in the whole cohort and UAP subgroup and a J-shaped correlation in the AMI subgroup, both with a nadir at PP 50–59 mmHg. (2) Cox proportional hazard model in the whole cohort showed that patients with admission PP <50 mmHg or PP ≥70 mmHg had a higher risk for long-term cardiac and all-cause death. (3) Further subgroup analysis confirmed the prognostic significance of PP <50 mmHg for cardiac and all-cause death or ≥70 mmHg for all-cause death in patients with AMI, but not in patients with UAP. This study suggested that PP measured at admission is a strong, independent prognostic marker predicting long-term mortality, especially in patients with AMI.

Previous studies mainly focused on the effect of SBP and DBP on cardiovascular prognosis, and most clinical practice guidelines recommended a blood pressure target of <140/90 mmHg in elderly patients and <130/80 mmHg in patients with DM or CAD (15, 16). However, there were few studies to expound on the optimal PP level for elderly patients with T2DM admitted for ACS. PP is the difference between SBP and DBP and approximately half of DBP. Physiologically, SBP and DBP increase with age in a parallel manner, but DBP beyond the age of 50–60 years tends to plateau and even decreases with increasing age, resulting in a large increase in PP after the age of 60 years (17). As a result, PP may be important as a specific risk factor in older individuals for cardiovascular disease (CVD). It is of note that patients with DM resemble older subjects regarding the predictability of PP for CVD because the aging of blood vessels begins at an earlier age in this population. This phenomenon could be explained by the pathophysiological finding that the formation of advanced glycation end products (AGEs), which play an important role in the development of atherosclerosis, is promoted by both aging and hyperglycemia (18, 19). Accumulation of AGEs on the protein of the extracellular matrix causes the formation of cross-links, which trap other local macromolecules. Cross-linking of AGEs on collagen and elastin increases the extracellular matrix area which makes the collagen insoluble to hydrolytic enzymes (20) and increases the stiffness of the artery (21). Besides, other mechanisms such as impaired glucose tolerance, reduced nitric oxide, increased endothelin-1, and abnormal neuroendocrine signaling may be involved in arterial stiffness by AGEs (18).

In the previous study, PP was considered an independent cardiovascular risk factor in various populations. Increased PP appears to be the best single measure of blood pressure in predicting mortality in older people aged 65–102 years (22). A study including 3,120 patients with T2DM and/or hypertension showed that patients in both PP <45 mmHg and PP > 55 mmHg groups were at increased risk of future coronary heart disease, and the association is independent of SBP, DBP, or mean arterial pressure (MAP) (23). SAVOR-TIMI 53 trial showed that increasing PP was associated with a higher risk of MI and ischemic stroke for patients with T2DM (24). Kodama et al. performed a quantitative meta-analysis to estimate CVD risk in relation to 4 commonly used BP indexes (i.e., PP, MAP, SBP, and DBP) in patients with DM (25). The results suggested that PP was the strongest indicator and might be the most accurate factor for estimating CVD risk.

Generally, high PP has been shown to be superior to low PP as a useful predictor of adverse cardiovascular prognosis, especially in older people, and it is unknown whether these associations are modified among elderly patients with T2DM admitted for ACS. In fact, low PP may help identify patients at high risk due to severe left ventricular dysfunction and decreased stroke volume. A study enrolling 6,704 consecutive patients with ACS found that low PP was an independent predictor of stroke and mortality in the overall cohort. Although PP was not superior to SBP, only low PP was an independent predictor for recurrent ischemia in non-ST-segment elevation ACS (NSTE-ACS) (7). Tan et al. found that lower PP was independently associated with in-hospital mortality after adjustment for other GRACE risk model predictors among patients with ACS (26). Conversely, an analysis from Special Program University Medicine-Acute Coronary Syndrome study showed that, for every 10 mmHg increase in PP, there was a 13% increase in all-cause mortality and more than a 20% increase in recurrent MI after ACS (27). Unlike these studies performed in a broad spectrum of patients with ACS, our study included elderly patients with T2DM and ACS. In the whole cohort, the patients with the lowest PP (<50 mmHg) or highest PP (≥70 mmHg) all had higher cardiac and all-cause death during long-term follow-up when compared with patients in PP 50–59 mmHg group. A U-shaped correlation was seen in this study for admission PP and cardiac or all-cause death with a nadir at 50–59 mmHg. After adjustment, PP <50 mmHg was still an independent predictor for cardiac and all-cause death, whereas PP ≥70 mmHg only exhibited an increased risk for all-cause death. These findings that are different from some previous studies suggested that the prognostic value of admission PP may exhibit discrepancies with respect to different populations or ACS types.

The physiological mechanism and prognosis predictors of UAP and AMI are not completely consistent. Less is known about the difference in the prognostic implication of PP in these two subtypes of ACS, especially for elderly patients with T2DM. In this study, U-shaped and J-shaped correlations were seen between admission PP and cardiac or all-cause death during follow-up in UAP and AMI subgroups, respectively, with a similar nadir at 50–59 mmHg. Even more importantly, after adjusting for the potential confounding factors, PP no longer independently predicted follow-up cardiac and all-cause death in the UAP subgroup, although PP <50 mmHg showed a trend toward increased risk for all-cause death. Nevertheless, PP remained to have a predictive value for mortality in the AMI subgroup. When compared with patients in PP 50–59 mmHg group, patients in PP <50 mmHg group still had higher risk for cardiac death (HR 2.92, 95% CI 1.45–5.76, *P* = 0.002) and all-cause death (HR 2.08, 95% CI 1.20–3.58, *P* = 0.009), respectively. In addition, PP ≥70 mmHg was only associated with a higher risk of all-cause death (HR 1.78, 95% CI 1.05–3.00, *P* = 0.031). Our findings were not completely consistent with most previous reports. In 1997, Mitchell et al. evaluated the relationship between PP, measured 3–16 days after MI, and subsequent adverse events in 2,231 patients with left ventricular dysfunction. The study reported that high PP was a significant predictor of total mortality (28). Ma et al. performed a retrospective study on 7,033 patients with consecutive STEMI and found that PP <40 mmHg had greater cumulative mortality within 30 days (29). There was a pattern of declining HRs with increasing blood pressures for average SBP and PP but not for DBP or MAP, and this trend was mainly observed in patients aged more than 70 years old. However, these patients were mainly treated by thrombolysis instead of PCI in our study. Other studies have also associated low PP with increased mortality (6, 7), but these associations were greatly driven by patients with severely decompensated heart failure or cardiogenic shock. To avoid these confounders, we excluded patients who had severe pump failure with admission SBP <90 mm Hg or Killip class IV. Ultimately, low PP was still prognostically significant even after adjusting for many traditional and important potential confounders including age, gender, and LVEF.

Despite advances in medication and revascularization strategies, mortality remains high after ACS. Currently, it is mainly SBP and DBP that are included in prognostic risk scores and addressed by guidelines, whereas no specific recommendations concerning PP have been proposed. Our study fulfills the research gap regarding the prognosis value of PP among elderly and diabetic patients in the setting of ACS and reinforces that PP should be considered in the development of clinical prediction models and the establishment of the blood pressure goal. The findings may assist clinicians in deciding about the intensity of blood pressure control for the growing cohort of older adults with multiple chronic conditions.

Some limitations in our study need to be mentioned. First, this study is a single-center, retrospective observational study, and the reported relationship applies to PP measured in admission for ACS. Thus, the generalization of the findings should be cautious. Second, our study could not prove a clear effect of PP but suggested a possible prognostic marker. The underlying mechanisms for the discrepancy in the association between mortality and admission PP in different populations are not completely clarified, may be multifactorial, and cannot be extrapolated from our observational findings. Third, we measured PP in the peripheral, which does not perfectly match central PP due to pulse wave amplification. Finally, our analyses did not adjust for some potential confounders in the elderly, such as frailty, socioeconomic status, and mental health, which may be associated with adverse outcomes. Besides, diabetes and prediabetes are associated with an increased risk of developing heart failure and cardiovascular event recurrence (30), but other major cardiovascular outcomes except death were not included in this study.

## Conclusions

In elderly patients with T2DM admitted for ACS, PP measured at admission is a strong, independent prognostic marker of long-term cardiac and all-cause mortality, especially in patients with AMI. There is a U-shaped correlation between PP and risk for mortality in UAP and a J-shaped correlation in AMI, both with a nadir at 50–59 mmHg. Measuring PP may contribute to the evaluation of the individual risk and therapeutic decision-making, and PP should be considered for the management of secondary prevention. Further prospective cohort studies are needed before proposing PP as a target for therapy.

## Data Availability Statement

The raw data supporting the conclusions of this article will be made available by the authors, without undue reservation.

## Ethics Statement

The studies involving human participants were reviewed and approved by Ethics Committee of Beijing Friendship Hospital affiliated to Capital Medical University. The patients/participants provided their written informed consent to participate in this study. Written informed consent was obtained from the individual(s) for the publication of any potentially identifiable images or data included in this article.

## Author Contributions

ZW and WL performed study, statistical analysis, and wrote manuscript. YW, BB, and XD participated in study data collection. XL contributed discussion and edited manuscript. HL and WL provided funding support, designed study, and reviewed manuscript. All authors have read and approved the final manuscript.

## Funding

This study was supported by National Key R & D Program of China (No. 2021ZD0111004), National Natural Science Foundation of China (Grant No. 82070357), Beijing Municipal Administration of Hospitals Incubating Program (Grant number PX2018002), and Beijing Key Clinical Subject Program.

## Conflict of Interest

The authors declare that the research was conducted in the absence of any commercial or financial relationships that could be construed as a potential conflict of interest.

## Publisher's Note

All claims expressed in this article are solely those of the authors and do not necessarily represent those of their affiliated organizations, or those of the publisher, the editors and the reviewers. Any product that may be evaluated in this article, or claim that may be made by its manufacturer, is not guaranteed or endorsed by the publisher.
